# 

**Published:** 1854-06

**Authors:** 


### INDEX TO VOLUME TENTH.

*** For original communications, look for the names of the authors as well as the titles of the subjects.

For selected articles look for the titles of the subjects.

☞ For notices of books look for names of authors.

A — PAGE.

Abel and Bloxam's Chemistry,..... 87
Abortion, means of, ................ 285
Abscess, treatment of milk, ........ 165
Address of Dr. Parsons to American Medical Association, ............. 65
" of Dr. Hunt to Erie Co. Medical Society, ...................... 147
" of Dr. Hunt to Graduates of Med. Dep. Buff. University,........... 577
American Medical Association, meeting of,........................ 48
" Publication, committee of,..... 114
" Transactions of,.......... 445, 464
American Medical Society of Paris,. 309
Amputation in a case of Gangrene. By Drs. Jameson and Avery,..... 397
Anchylosis, treatment of,.......... 90
Anatomical Specimens, preservation of, .......................... 162
Anaplasty applied to treatment of old Ulcers. By Frank H. Hamilton, M. D.,.................. 433
Anæsthesia, local,................ 573
Apologies, ...................... 382
Arink's Medical Manual, .......... 540
Atlee on Blood,.................. 427
Avery, Drs. Jameson and, case of amputation,..................... 387
" Drs. Jameson and, on Erysipelas, 355

B

Barton, Prof. Edward H., Report on Sanitary conditions of New Orleans,.................. 497, 537
Baker, Charles H., M. D., on Occlusion of Vagina,.................. 37
" on Epilepsy, ................. 73
Baths, Devergie on. Translated by Prof. Flint, ............... 449, 525
Bernard on Blood, ................ 427
Beck on Infant Therapeutics, ...... 541
Bellows, M. D., Matthias B., death of, 188

PAGE.

Bigelow's Nature in Disease,....... 481
Blood, circulation of, .............. 97
Boston, changes in,................ 633
Buffalo Med. Association. Minutes of meetings, ............. 37, 205, 405
" " " Proceedings of, 715
Buffalo University. Medical department of the,.................. 300, 571
" " " Commencem't of, ........................... 634
" " " Graduates in,. 536
Buffalo, Health of,............ 373, 442

C

Camphor an antidote to Strychnia,.. 572
Carcinoma Uteri, case of. By John G. House, M. D.,................. 271
Carpenter's Comparative Physiology 273
" Alcoholic Liquors,...... 86
Cartwright on Drapetomania,...... 438
Carbuncle,....................... 679
Cholera,. 180, 248, 384, 447, 570, 621, 698
" Clericus, Jr., on, .......... 304
" Sulph. Acid in. By N. L. North, M. D.,..................... 167
" Congestion of Brain in. By James M. Newman, M. D.,....... 333
Channing, Prof. Walter, ........... 312
Chloroform,....................... 313
Clairvoyant,...................... 383
Clericus, J., a Short Sermon by,.... 70
" " on Cholera,............ 344
Counter-Poisons, .................. 314
Constituents of Food. By Sanford B. Hunt, M. D.,................... 455
Consumption. Sugar-house cure,.. 511
Coroner's Inquest,................. 574
Collapse of Lung. By Sanford B. Hunt, M. D., .................... 591
Crandall, C. Millford. Case of Rupture of the Uterus,.............. 663
Conjunctiva, Granular, ............ 700

PAGE.
Correction, 703
Croup, 673

D
Dalton on Gastric Juice, 553
Devergie on Baths. Translated by Prof. Flint, 449
Dental Works, 477
Dissections, Judicial, 559
Dislocation of Vertebra, 702
" of Hip-joint, cases treated by Reid's method, 604
Drapetomania, 438
Drake on the Principal Diseases, 425
Dysentery, Parotitis in, 315

E
Epilepsy. By Chas. H. Baker, M. D., 73
" Tracheotomy in, 443
Epidemic causes, 181, 311
Elkoplasty applied to the treatment of old ulcers. By F. H. Hamilton, M. D., 433
Erichsen's Surgery, 87
Ergot, 286
Erysipelas. By Drs. Jameson and Avery, 355
Eruptive Disease in Hornellsville. By W. H. Reynale, M. D., 385
Ether in Surgery. By Charles Mayor, M. D., 522
Exsection of Superior Maxilla. By H. A. Potter, M. D., 589
Explanation, 622

F
Fatty Degeneration, 216
Fevers, remedy for, 165
Fever, Intermittent, 672
" Typhoid. By Edward R. Maxson, M. D., 68
Fractures, Ununited, 510
" of Humerus. By Frank H. Hamilton, M. D., 142
" of Skull, 686
Fuller on Rheumatism, 150, 470
Femoral Artery, ligation of. By F. H. Hamilton, M. D., 266
Flint, Austin, M. D., on Chronic Pleurisy, 1
" Letters from Paris, 107, 129, 192, 321, 412
" Translation of Devergie on Baths, 449, 525
" Valedictory,

G
Galvanism in Obstetrics, 286
Gastric Juice. By J. C. Dalton, M. D., 553

PAGE
Gout, gentlemanly, 448
Gross on Foreign Bodies, 664
Green on Injection of Tubercular Cavities, 693
" Letter from Prof. Horace,
Griscom's Anniversary Discourse to N. Y. Academy of Medicine, 695

H
Hamilton, Prof. F. H., on Fractures of the Humerus, 142
" Elkoplasty, 433
" Ligation of Femoral Artery above Profunda, 266
" Hyatts' collection, 367
" Reply to Dr. Reynale on Hornellsville Epidemic, 209
Hall, Excito-moter system of, 174
Hassall's Microscopic Anatomy, 478
Harrison's Anatomy, 541
Heywood, John Wicks, M. D., death of, 63
Hendrickson's Trial, Lee's review of, 505
Hequembourg, Rev. C. L., on the existence of a true interparietal bone in some human races, 513
Hernia, 575, 675
Hip-joint, dislocations of, 604
Hornellsville Epidemic, F. H. Hamilton, M. D., on, 209
" W. H. Reynale, M. D., on, 385
House, M. D., John G., on Carcinoma Uteri, 271
Holmes on Puerperal Fever, 595
Howell, M. D., William. Thesis on the Liver as a sugar producer, 654
Hubbard, M. D., Silas, on Theories of the Production of Sexes, 645
Hysteria, S. B. Hunt, M. D., on, 22
" Treatment of, 169
" Supposed, 216
Hydrorrhœa, 213
Hyatts' Collection. By Frank H. Hamilton, M. D., 367
Hunt, M. D., Sanford B., on Hysteria, 22
" Powers of Human Races, 147
" Constituents of Food and Drink 455
" Valedictory Address to Graduates in Medicine, 577
" Collapse of Lung, 591
" Meteorological Tables, 77, 177, 198, 277, 369, 430, 482, 542, 601, 669, 726

I
Insanity, Earle on, 248
" Institutions for, 249
Iodine, 316
Interparietal Bone, on the existence of. By Rev. C. L. Hequembourg, 513
Iodide of Iron. By Richard W. Nelson, M. D., 642

### J

PAGE.

Jameson and Avery, on Epidemic Erysipelas, ... 355
" " " on case of amputation, ... 397
Jones and Sieveking's Pathological Anatomy, ... 668

### K

Kolliker's Microscopical Anatomy, ... 429

### L

Letters from Paris, by Prof. A. Flint, 107, 129 193, 257, 321, 412
Lee, M. D., Charles A., on Statistics of Medical Profession, ... 203
" on trial of Hendrickson, ... 505
Liver as a Sugar Producer. Thesis by Wm. Howell, ... 654
London, Health of, ... 301
List of Contributors to Vol. X., ... 753

### M

Meteorological Table. By Sanford B. Hunt, M. D., 77, 177, 198, 277, 369, 430, 482, 542, 601, 669, 726
" of California. By T. S. McLellan, ... 721
Maxson, Edwin R., M. D., on Typhoid Fever, ... 68
" on Rheumatic Fever, ... 268
" new Respiratory Sound, ... 587
Meigs on Woman, ... 82
" on Childbed Fevers, ... 419
Medical Profession, statistics of. By C. A. Lee, M. D., ... 203
Menstruation. Its effects on milk, ... 284
Mayor, M. D., Charles, on Uses of Ether in Surgery, ... 522
Malpractice, trial for, ... 566
Milk Solidified, ... 381
Michigan University, ... 630
Mortality of Buffalo, tables of. By James M. Newman, M. D., 47, 88, 160, 211, 278, 370, 432, 483, 543
Mortality of Buffalo, tables of. By John Root, M. D., ... 602, 670, 724
Mortality of New York, ... 187
Moore, Prof. E. M., ... 250
Morbus Coxarius, ... 544
McLellan. Meteorology of California, 721
Monstrosity, ... 737

### N

Newman, M. D., James M., on Mortality of Buffalo, 47, 88, 160, 211, 278, 470, 432, 483, 543
" on Congestion of the Brain in Cholera, ... 333
" on Vaccination, ... 458
" Sanitary report of, ... 639
" Review of Mass. Registry Law, 705
Night Sweats in Phthisis, ... 164

PAGE.

Nelson, M. D., Richard W., on Iodide of Iron, ... 642
Novelties, therapeutical, ... 381
Nott and Gliddon, Types of Mankind 40
North, M. D., N. L., on Sulph. Acid in Cholera, ... 400

### O

Obituary of J. W. Heywood, M. D., ... 63
" of Amatus Robbins, M. D., 188
" of M. B. Bellows, M. D., ... 188
" of Prof. Swett, ... 283
" of Mrs. Eliza Rogers, ... 252
Oration before Physico-Medical Society of New Orleans. By A. Mercier, M. D., ... 696
Ourselves, ... 575
Œsophagotomy, ... 558
Opium, ... 279
Ovarian Cyst, ... 680

### P

Parsons, M. D., Usher, address by, ... 65
Paracentesis, Thoracic, ... 95
Parturition, sudden death in, ... 283
Peaslee, Prof. E. R., Review of Fuller's Rheumatism, ... 470
Pleurisy, Chronic, cases of. By Austin Flint, M. D., ... 1
Potter, M. D., H. H. Exsection of Superior Maxilla, ... 589
Powers of Human Races, on. By S. B. Hunt, M. D., ... 147
Poorhouse of Erie County, ... 184
Principles of Physiology, ... 480
Puerperal Peritonitis, ... 697

### R

Ramsbotham's Midwifery, ... 668
Respiratory Sound, new. By Edwin R. Maxson, M. D., ... 587
Reid's method for Dislocation of the Femur, ... 604
Report of N. Y. Lunatic Asylum, ... 628
" " Indiana, ... 636
Reynale, M. D., W. H., Prof. Hamilton's Reply to, ... 209
Rheumatic Fever. By Edwin R. Maxson, M. D., ... 268
Ringworm, ... 93
Rogers, Mrs. Eliza, death of, ... 252
Root, M. D., John M. Mortality of Buffalo, ... 602, 670, 724
Report of Births, Marriages and Deaths in Mass. Review by J. M. Newman, M. D., ... 705

### S

Sanitary Report of New Orleans, 497, 537
" " Buffalo, ... 639
Salt, ... 313

PAGE.

Scapula, excision of, ............... 91
Sermon. By Clericus, Jr., .......... 70
Serpent Bites, .................... 657
Simaba Cedron, .................... 672
Smithsonian Institution, ...... 306, 561
Spirit Rappings, .................. 241
Stethoscope, Cammann's, ........... 701
Sulphuric Acid in Cholera. By N. L. North, M. D., ............... 400
Swett, Prof., death of, ........... 383
Syphilis, nitric acid in, ......... 166
Surgery of the War, ............... 691
Stick in the Stomach, ............. 689

T

Theories of the Production of the Sexes. By Silas Hubbard, M. D., 642
Tongue in Diagnosis, .............. 484
Tracheotomy, ...................... 443
Transactions of Medical Society of Erie County, .......... 117, 532, 565
" of Southern Central, N. Y., .... 250
" State Society, N. Y., .......... 623
" " " Illinois, ........ 636
" " " N. Hampshire, ... 696
" " " Pennsylvania, ... 446
" N. Y. Pathological Society, .... 618
" American Med. Association, 445, 464

PAGE.

Types of Mankind, ................. 40
Tampon, Trousseau's, .............. 252

U

Ulceration of Legs, ............... 681
Utilitarianism, ................... 253
Uterus, Rupture of, ............... 487
" " " case of. By C. Milford Crandall, M. D., .......... 663

V

Vagina. Occlusion of. By C. H. Baker, M. D., .................... 37
Vaccination. By James M. Newman, M. D., ...................... 458
Version, Spontaneous, ............. 92
Vidal on Venereal, ................ 159
Viviparous Fish, .................. 484
Vertebral Dislocation, ............ 702
Valedictory to the Readers of Buff. Med. Jour. By Senior Editor, .... 750

W

Water Cure, pleasures of, ......... 252
West on Diseases of Children, ...... 85
" " Os Uteri, .................. 665
What to Observe, .................. 667
Wound, Bayonet, ................... 560

